# Microstructural Modeling and Strengthening Mechanism of TiB/Ti-6Al-4V Discontinuously-Reinforced Titanium Matrix Composite

**DOI:** 10.3390/ma12050827

**Published:** 2019-03-11

**Authors:** Shuai Zhao, Yangjian Xu, Changliang Pan, Lihua Liang, Xiaogui Wang

**Affiliations:** College of Mechanical Engineering, Zhejiang University of Technology, Hangzhou 310014, China; 13165983889@163.com (S.Z.); changliangpan666@163.com (C.P.); lianglihua@zjut.edu.cn (L.L.)

**Keywords:** titanium matrix composites, Thiessen polygon, microstructural modeling, strengthening mechanism

## Abstract

A novel modeling method was proposed to provide an improved representation of the actual microstructure of TiB/Ti-6Al-4V discontinuously-reinforced titanium matrix composite (DRTMC). Based on the Thiessen polygon structure, the representative volume element (RVE) containing the complex microstructures of the DRTMC was first generated. Thereafter, by using multiple user-defined subroutines in the commercial finite element software ABAQUS, the application of asymmetric mesh periodic boundary conditions on the RVE was realized, and the equivalent elastic modulus of the DRTMC was determined according to the homogenization method. Through error analyses on the experimental and calculated results regarding the equivalent elastic parameters of the DRTMC, the rationality of generating the DRTMC finite element model by using the present method was validated. Finally, simulations based on four types of network-like models revealed that the present simplifications to the particle shape of the reinforcement phase had less of an influence on the overall composite strength. Moreover, the present study demonstrates that the DRTMC enhancement is mainly attributed to the matrix strengthening, rather than the load-transferring mechanism. The strengthening influences of the distribution forms of the reinforcement phases, including their distribution density and orientation, were studied further. It was found that both the higher distribution density and limited distribution orientation of the particles would increase the probability of overlapping and merging between particles, and; therefore, higher strength could be yielded when the volume fraction of the reinforcement phase reached a certain threshold. Owing to the versatility of the developed methods and programs, this work can provide a useful reference for the characterization of the mechanical properties of other composites types.

## 1. Introduction

With the development of the aerospace industry, the requirements for the adopted materials have become more focused on lightweight, but high strength, ductility, and toughness. In order to meet the development trends, studies on lightweight titanium alloy and composites have become an important direction in material development, owing to their high performance and lightweight features. In particular, discontinuously-reinforced titanium matrix composite (DRTMC), which not only maintains the excellent properties of titanium alloy, but also possesses a higher specific strength and specific modulus, is expected to become a key structural metal material in the aerospace field. Therefore, this area has received increasing attention in material research in recent years [1,2].

The ex-situ and in-situ methods are popular for the material preparation of the DRTMC. At present, a significant number of research achievements have been gained in its preparation process, the evolution of its microstructure, and the characterization of its material properties. Experimental tests have demonstrated that a composite with discontinuously-reinforced distribution, compared to uniform distribution of its reinforcement phase, is superior in terms of strength and plasticity. Two main explanations can be provided for such an enhancement of material properties: the load-transferring and matrix-strengthening mechanisms. The high strength and roughness of TiB whiskers can improve the overall mechanical performance through the load-transferring mechanism, while the refinement of the metal matrix, resulting from in-situ reaction and a solid solution, among others, will also contribute a strengthening effect. Certain researchers have stated that the strengthening of the DRTMC is mainly owing to the load-transferring mechanism. For example, Li et al. [3] studied the strengthening effect of TiC–TiB reinforced composites at room and high temperatures from the perspective of the load-transferring mechanism. Chandravanshi et al. [4] found that the creep resistance of titanium alloy increases with an increasing reinforcement phase content. The authors declared that this could be owing to the strengthening caused by non-deformed whiskers. Das et al. [5] considered that TiB whiskers reinforce the composite by means of the load-transferring mechanism, owing to the strong interfacial bonding between the TiB, formed in situ, and the titanium matrix. However, other researchers stated that reinforcement of the matrix material plays a dominant role. For example, Wang et al. [6] simulated the mechanical response of B_4_C hybrid-enhanced discontinuously-reinforced titanium matrix composites (DRTMCs) based on a yield strength model, by accounting for strengthening from the reinforcement phase, matrix grain refinement, and solid solution simultaneously. Their results indicated that the increase in the composite yield strength is mainly owing to grain refinement and solid solution strengthening. Zherebtsov et al. [7] demonstrated that the contribution of the Hall–Petch hardening and substructure hardening mechanisms expectably increase with the strain, owing to the microstructure refinement and increase in dislocation density. Guo et al. [8] believed that the grain refinement and reinforcement phase tilting were the main reasons for the increase in the composite yield strength, by means of studies on the grain refinements of the TiB reinforcement phase, La_2_O_3_ particles, and matrix. Wang et al. [9] analyzed the DRTMC using both the two-step and direct methods, and proved that the material strengthening mechanism can mainly be attributed to the grain refinement and solid solution strengthening of the metal matrix. The authors believed that the load-transferring mechanism has little effect on the material strengthening. The literature review demonstrates that the fundamental cause regarding the DRTMC strengthening mechanism remains unclear, and different reinforcement phases may derive different conclusions.

At present, numerical investigations on the DRTMC are scarce. Although it has frequently been used in studies on metal matrix composites, most of the modelling of Ti6Al4V alloy is still relatively simple. Aradhya and Doddamani [10] evaluated the specific strain hardening behavior of SiC/Ti-6Al-4V by constructing a simple fibrous model. Giannopoulos et al. [11] designed a kind of periodic whisker arrangement model to analyze the mechanical behavior of Ti64 composites by use of finite element method (FEM). De et al. [12] modeled a thermo-fluid dynamical model to investigate the evolution of the microstructure and temperature profile. By modeling polygonal network reinforcement architecture, Wang et al. [9] predicted the elastic properties and revealed the strengthening mechanisms of DRTMC. In short, the reinforcement phases have generally been modeled by using particles with simple shape, and their reinforcement phase distributions were also quite different from the actual situation.

With the aim of addressing the above issues, this paper firstly introduces a novel microstructure modeling method based on the Thiessen polygon and its programming-related details. Thereafter, the application of asymmetric mesh periodic boundary conditions (AMPBCs) on the representative volume element (RVE) in the commercial finite element software (ABAQUS), through user-defined subroutines, is illustrated, as well as the homogenization method. Furthermore, the calculated equivalent elastic parameters of the DRTMC are used to verify the effectiveness of the developed methods. Subsequently, the simulation results of simplified network-like distributed models, accounting for a micro-mechanism material constitutive relationship are investigated in comparison with the experimental results. Moreover, the influences of simplifying the reinforcement phase and strengthening mechanism of the DRTMC materials are disclosed. Finally, the effects of the reinforcement phase distributions, including the density and orientation, on the mechanical properties of the composites are investigated, as well as the influence on simplification from a three-dimensional (3D) to two-dimensional (2D) model.

## 2. Modeling of Network-Like Microstructure

### 2.1. DRTMC Microstructure

As an objective for improving both toughness and strength, DRTMC is designed as a titanium-based composite, with quasi-continuous network-like distribution of its reinforcement phase. This TiB whisker reinforced composite (TiB/Ti-6Al-4V) consists of large-size titanium alloy powder and fine TiB_2_ powder, prepared by an in-situ reaction incorporating the powder sintering technique. It not only possesses high strength, but also maintains effective performance in terms of toughness. Owing to the excellent mechanical properties, its network-like enhanced microstructure has attracted significant attention.

**Figure 1:**
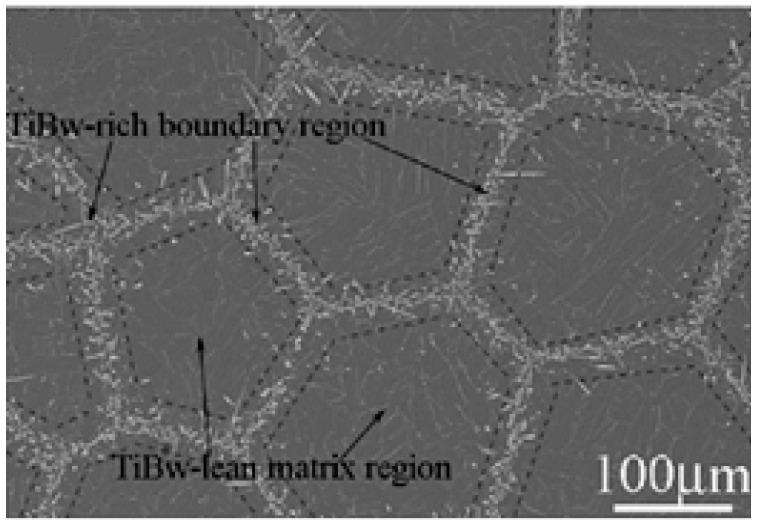
Micrograph of network-like reinforced TiB/Ti-6Al-4V composite [13].

Figure 1 illustrates the network-like enhanced microstructure of the DRTMC. As can be observed from this figure, the TiB reinforcement phase is randomly distributed at the boundary of the cells, which are made of Ti-6Al-4V with a diameter of approximately 200 μm. Therefore, the entire microstructure can be divided into two regions: The central reinforcement-lean region of the matrix material, and the reinforcement-rich region, similar to the interface between cells, which are denoted as Phase-I and Phase-II in this context, respectively. It is worth noting that the Phase-I region is nearly pure matrix material, with few whiskers existing.

### 2.2. Microstructure Modeling Method

The shape, size, and spatial distribution of the Phase-II region in the DRTMC microstructure exhibit a certain degree of randomness. In order to provide an improved representation of the DRTMC microstructure, a realistic modeling method was developed in our work, based on the algorithm of shrinking the Voronoi cells (Thiessen polygons) [14], which was developed by scholars [15,16]. By means of Fortran programming, the network-like microstructure model containing Phase-I and Phase-II can be generated automatically, and finally transferred to form a 2D RVE finite element model for mechanical simulation. The detailed method and main steps are summarized as follows:

(1) As the network-like microstructure of the DRTMC is very similar to the Thiessen polygon, we proposed simulating the network-like structure of Phase-II using Thiessen polygons. The essential principle and method for generating Thiessen polygons that were used to describe the network-like structure of the studied material are generalized here.

Firstly, let $A=\left\{a_{1},a_{2},\cdots ,a_{n}\right\},\left(2<n<\infty \right)$ be the point set on the Euclidean plane *K*^2^, and for ∀i,j={1,2,⋯,n}, if i≠j, there must exist $a_{i}\neq a_{j}$. In this paper, the distance between two points *a_i_* and *b* is represented by the Euclidean distance *d*(*a_i_*, *b*).

The Thiessen region associated with *a_i_* is given by Equation (1):

(1) $$V\left(a_{i}\right)=\left\{b\in K^{2}|d\left(a_{i},b\right)\leq d\left(a_{j},b\right),j\neq i,j\in I_{n}\right\},$$

where *I_n_* is a set of integers from 1 to *n*.

Thereafter, the Thiessen diagram, or alternatively, a Voronoi diagram [17], generated by *a_i_*, is given by the set

(2) $$V\left(A\right)=\left\{V\left(a_{1}\right),V\left(a_{2}\right),\cdots ,V\left(a_{n}\right)\right\}.$$

According to the micrograph of the DRTMC microstructure, as indicated in Figure 1, the geometrical information with respect to the size and shape of the cell-like Phase-I region can be obtained statistically. By controlling the distribution of point *a_i_* in the above equations, the network-like shapes can be enriched and finally be approximated to the actual microstructures. For example, the average network-like sub-region size in Figure 1 is approximately equal to 200 μm. By changing the number of network-like sub-regions and adjusting the outer boundary size, similar sizes of cells can be produced.

**Figure 2:**
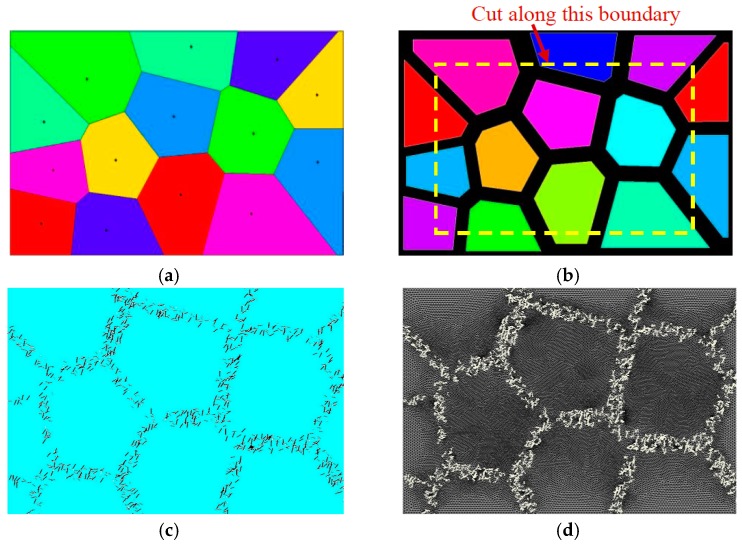
Generation process of discontinuously-reinforced titanium matrix composite (DRTMC) finite element model: (**a**) generating Thiessen polygons; (**b**) generating placing region of reinforcement phase; (**c**) placing particles of reinforcement phase; and (**d**) model meshing.

**Figure 3:**
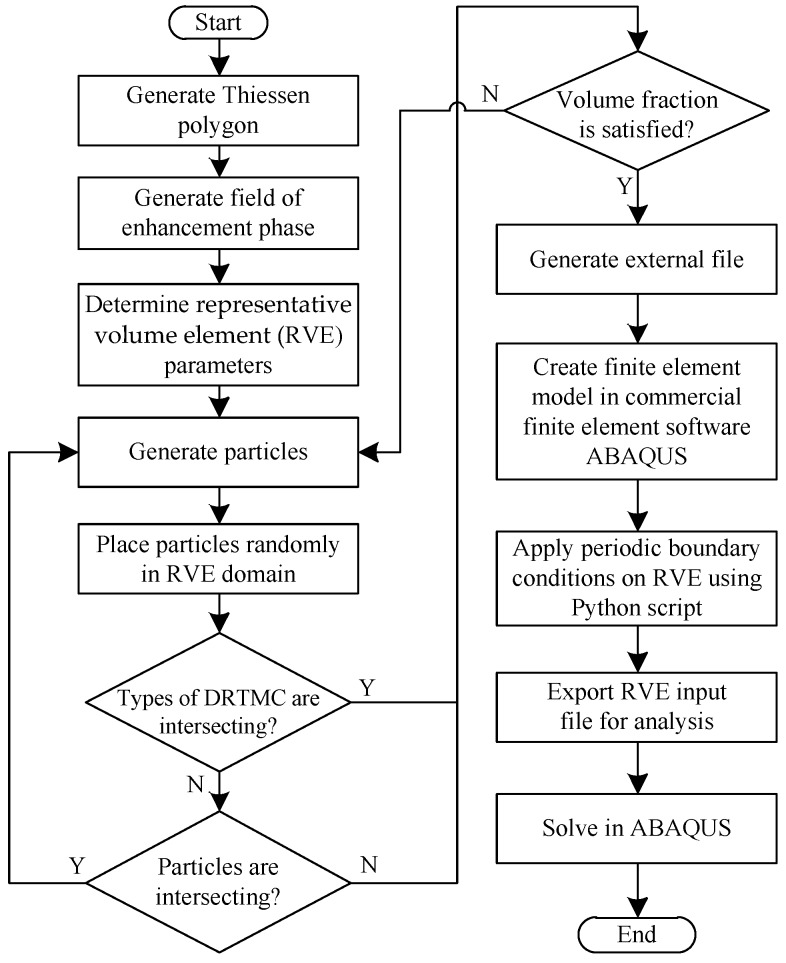
Flowchart of modeling and simulation of DRTMC microstructures.

(2) At the corner of the Thiessen polygon, *n*−1 control nodes were replicated at the same location, according to the number (*n*) of sub-regions connected to the corner. Then, the distribution region of the reinforcement phase (Phase-II) was generated by the associated algorithm, as illustrated in Figure 2. The average bandwidth of the distribution region for Phase-II was statistically obtained from Figure 1. Because the width of each band differs, a realistic width for each region was prescribed through a random function, based on the mean width.

(3) Once the distribution regions of Phase-I and Phase-II were defined, the related spatial geometrical information of these regions was exported to an external file. By using a Fortran routine, the related information in the file was further extracted, and the reinforcement phase with various shapes (such as circles, squares, and triangles) was randomly generated and placed in the Phase-II region. During placement of the reinforcement phase, its volume percentage was controlled according to the actual situation. In our work, two placing schemes for the reinforcement phase (that is, separated and intersected between enhanced particles) were considered. For the separated case, it was relatively easy to control the content of the reinforcement phase, but overlap detection between different particles was required. In order to facilitate generation of the finite element mesh of the microstructure, the minimum distance between particles must be larger than a prescribed threshold. For the intersected case, it was not necessary to repeat the placement of particles, as the particles could be intersected. Once the actual content of the reinforcement phase was reached, the placement of particles would be terminated. When the geometrical model of the microstructure was generated, the subsequent finite element modeling and simulation could be carried out. Figure 3 presents a flowchart with respect to the implementation of the microstructure modeling and finite element analysis for both cases. The present modeling method for the network-like microstructure can realistically represent the actual DRTMC microstructure and provide a guarantee for its accurate numerical simulation and analysis. Moreover, as the present method enables accounting for various shapes, contents, and spatial distributions of the reinforcement phase, it can be used to optimize the performance of this material type. Furthermore, it is suitable for characterizing other composite types.

In our work, the DRTMC microstructures were numerically modeled according to the above steps. Several reinforced particles distributed in the Phase-I region were not considered, as they had a negligible effect on its overall mechanical performance. Moreover, owing to the complexity of the reinforcement phase, three types of simplified particle shapes, namely separated circular, intersected circular, and rectangular particles, were investigated. For ease of narration, the different volume fractions (2%, 3.5%, and 5%) of the reinforcement phase in the TiB/Ti-6Al-4V materials are denoted by V2, V3.5, and V5, respectively. In order to ensure the reliability of the simulation results, at least five random configurations were simulated for each volume fraction, and finally, the average result was used for discussion and analysis.

## 3. Simulation and Analysis Based on RVE

### 3.1. Material Description of DRTMC

The selection of an appropriate material constitutive relationship plays a crucial role in properly simulating the mechanical responses of composites. If the matrix material does not contain any reinforced phases, that is, only Ti-6Al-4V is considered, its material constitutive relationship is relatively simple and generally written in the following form [18,19]:

(3) $$\sigma =\sigma_{ref}f\left(\varepsilon^{p}\right)=A+K{\left(\varepsilon^{p}\right)}^{n},$$

where *σ_ref_*, *A*, *K*, *ε^p^*, and *n* denote the reference stress, initial yield stress, strain hardening coefficient, plastic strain, and hardening exponent, respectively. Table 1 displays the adopted material properties of the DRTMC in the simulation. The elastic modulus *E* and Poisson’s ratio *μ* of the monolithic Ti-6Al-4V were obtained directly from the literature [13], while parameters *A*, *K*, and *n* were obtained by fitting the stress–strain curve of the Ti-6Al-4V alloy [13]. The reinforcement phase material was assumed as behaving linearly elastic and bonding ideally with the matrix. The Young’s modulus has generally been taken as a value between 425 and 482 GPa in the literature [20,21,22]. In our work, it was taken as 450 GPa, which was measured by nanoindentation [20].

It has been established [18] that particle-reinforced composites are prone to exhibiting a significant particle size effect. However, classic plasticity theories have no intrinsic material lengths and cannot explain the observed size effects. In general, it is believed [18] that three possible mechanisms are responsible for such size effects, which are generalized as follows: (1) Orowan bowing strengthening; (2) strain-gradient hardening, attributed to geometrically necessary dislocations associated with non-uniform plastic deformation in small volumes; and (3) quench hardening, resulting from statistically stored dislocations generated to accommodate the mismatch in the thermal expansion coefficients of the particles and matrix. According to the description in [18], the present composite could mainly be influenced by the strain-gradient and quench hardening. Therefore, Equation (3) was further modified in our work to consider the above two hardening mechanisms for describing the composite.

In general, the increase in the initial yield stress $\Delta \sigma_{y}$ in the metal matrix material owing to quenching is written as [23,24,25]:

(4) $$\Delta \sigma_{y}=\alpha G\sqrt{6\left|\Delta CTE\right|\left|\Delta T\right|\frac{V_{f}}{1-V_{f}}\frac{b}{d}},$$

where *α* is a constant of order unity; *G* is the shear modulus of the metal matrix material; ΔCTE is the difference in the thermal expansion coefficients between the matrix and particles; ΔT is the maximum temperature change during the heat treatment (quenching); *V_f_* is the particle volume fraction; *b* is the magnitude of Burgers vector of the metal matrix material; and *d* is the particle size. Therefore, the modified material constitutive relationship when considering quench hardening can be expressed as

(5) $$\sigma =\sigma_{ref}f\left(\varepsilon^{p}\right)+\Delta \sigma_{y}=A+K{\left(\varepsilon^{p}\right)}^{n}+\Delta \sigma_{y}.$$

In the present TiB/Ti-6Al-4V composite, *α* is taken as 1, which is in the same order of magnitude as the paper [25]. The thermal expansion coefficients of TiB and Ti-6Al-4V are 5.9 × 10^−6^/°C and 9.0 × 10^−6^/°C [26], respectively. The temperature change is 900 °C [13], the magnitude of Burgers vector is 0.295 nm [27], and the particle size is 5 μm [13].

In order to consider the particle size effect owing to strain-gradient hardening, Gao et al. [28] developed the Taylor-based nonlocal theory (TNT), which has been implemented in the finite element method framework [18,29], and is widely used to characterize the material plastic behavior of metal matrix composites.

According to the TNT, the flow stress of the matrix material considering the geometrical necessary dislocation is given by Gao and Huang [28], which can be written as

(6) $$\sigma^{*}=\sqrt{\sigma^{2}+27\sqrt{10}\beta^{2}G^{2}\frac{b}{d}V_{f}^{\frac{1}{3}}\varepsilon^{p}},$$

where *β* is an empirical material constant, and 0.5 is used in this case [27]. The other parameters have been explained above. The present constitutive law used in the simulation of the DRTMCs was achieved by the user-defined material (UMAT) subroutine in ABAQUS.

### 3.2. Application of Asymmetric Mesh Periodic Boundary Condition (AMPBC) on RVE

For the RVE in the composite material, it is more reasonable to apply a periodic displacement boundary condition (Equation (7)) to make the homogenization calculation.

(7) $$u_{i}^{j+}-u_{i}^{j-}={\bar{\varepsilon }}_{ik}\left(x_{k}^{j+}-x_{k}^{j-}\right)={\bar{\varepsilon }}_{ik}\Delta x_{k}^{j},$$

where $u_{i}^{j+}$ and $u_{i}^{j-}$ denote the displacements of the points at the boundary, perpendicular to the *i* coordinate axis; the superscripts *j*+ and *j*− indicate the directions of displacement at two opposite edges; ${\bar{\varepsilon }}_{ik}$ are the average strains of the RVE; $x_{k}$ is the coordinate value of any point at the RVE boundary; and $\Delta x_{k}^{j}$ is the edge length of the RVE model.

**Figure 4:**
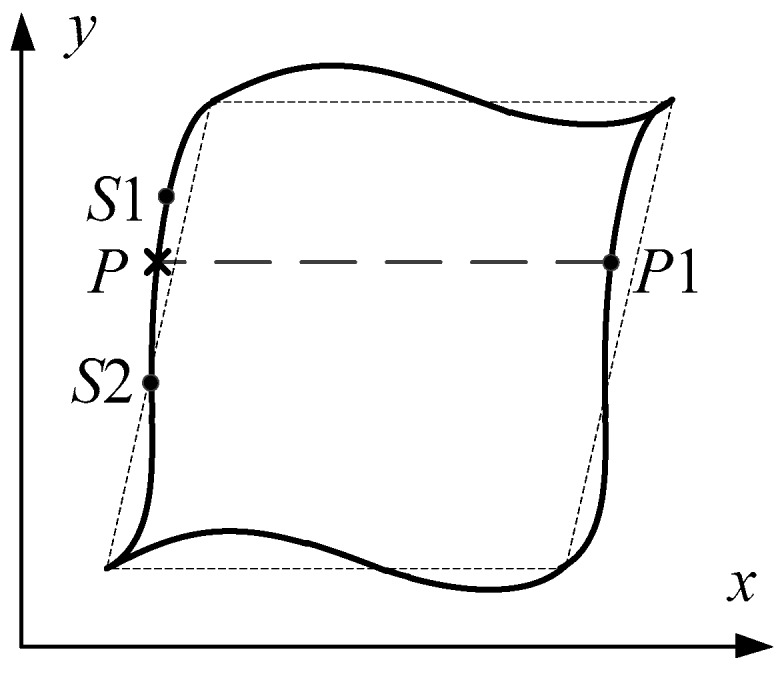
Schematic of interpolation calculation in nonconforming mesh of RVE.

It is relatively easy to impose the above boundary condition for the conforming meshes. Only two position-matched nodes at two opposite edges in an RVE model are involved during the application of periodic boundary conditions. For a nonconforming mesh, applying the periodic boundary conditions will no longer fulfill the one-to-one correspondence relationship at two opposite boundaries. Interpolation calculations between the nodes in the two opposite boundaries need to be conducted. For example, as illustrated in Figure 4, node *P*1 on the right boundary cannot find matched node *P* on the left boundary at an equal position. Therefore, the displacement of virtual point *P* should be calculated based on the interpolation from the nearest two nodes *S*1 and *S*2, as described by the following formula.

(8) $$u_{i}^{j-}\left(P\right)=N_{1}\left(P\right)u_{i}^{j-}\left(S1\right)+N_{2}\left(P\right)u_{i}^{j-}\left(S2\right),$$

where $u_{i}^{j-}\left(P\right)$ is the displacement of point *P*; *N*_1_(*P*) and *N*_2_(*P*) are the corresponding shape functions of points *S*1 and *S*2.

Combining Equations (7) and (8), the general form of the constraint equation for the AMPBC can be provided:

(9) $$u_{i}^{j+}\left(P1\right)-\left[\begin{array}{cc} N_{1}\left(P\right) & N_{2}\left(P\right) \end{array}\right]\left[\begin{array}{c} u_{i}^{j-}(S1)\\ u_{i}^{j-}(S2) \end{array}\right]={\bar{\varepsilon }}_{ik}\Delta x_{k}^{j},$$

where *i*, *j* = 1, 2. In our work, the application of periodic displacement boundary conditions was implemented in ABAQUS by means of the following linear multi-point constraint relationship.

(10) $$C_{1}u_{P}^{i}+C_{2}u_{Q}^{j}+\ldots +C_{n}u_{R}^{k}=0,$$

where *C_n_* is the correlation coefficient that corresponds to the displacement coefficient variables in Equation (9), and its subscript *n* is the number of terms correlated to the constraint relationship; the superscript of the displacement variable represents the degree of freedom, while its subscript denotes the node position. As the application of AMPBC on the RVE is quite onerous, codes written in Python scripts were used to achieve this operation automatically in our work.

### 3.3. Characterization of Equivalent Material Properties Using the Homogenization Method

The homogenization method has been popularly applied to characterize the equivalent material properties of heterogeneous materials through the RVE. In the present work, the homogenization method proposed in [30,31,32] was implemented in ABAQUS, through the associated user-defined subroutines, to achieve calculation of the equivalent material properties of the DRTMC. By means of the user-defined subroutine UEXPAN and the use of the thermal strain concept [31], three types of unit strain conditions can be imposed, and their results can be calculated. After completing the above calculations, the stress information at the integration points can be obtained through the user-defined subroutine URDFIL, following which the equivalent stiffness matrix can be calculated by means of the user-defined subroutine UEXTERNALDB, according to Equation (11).

(11) $$L_{mnpq}=\frac{1}{\left|\theta \right|}\int_{\sigma }\theta_{mn}^{pq}d\theta =\frac{1}{\left|\theta \right|}\sum_{i=1}^{n_{\mathrm{int}}}\sigma_{mn}^{pq}\left(i\right)J\left(i\right)W\left(i\right)$$

where |θ| is the RVE volume or area; *n*_int_ is the total number of integration points in the RVE finite element model; *J*(*i*) and *W*(*i*) are Jacobian determinants, and weight at integration point *i*, respectively; $\sigma_{mn}^{pq}\left(i\right)$ is the stress influence function, that is, stress induced by an overall unit strain $\varepsilon_{pq}$. In the calculation, different unit strain conditions $\varepsilon_{pq}$ are imposed on the RVE to calculate their respective stresses $\sigma_{mn}^{pq}\left(i\right)$ for each integration point *i*. Based on all the calculated stresses at different unit strain loading conditions, the equivalent stiffness matrix $L_{mnpq}$ can be finally calculated. The detailed explanation regarding this formula can be found in [31].

## 4. Verification of Modeling and Simulation Methods

Prior to applying the proposed modeling and simulation methods to investigate the DRTMC strengthening mechanism, these need to be verified. The identification of the equivalent elastic parameters of the DRTMC using our developed methods provides a good verification example. Here, the simulation process and a comparison of the results are briefly demonstrated.

Taking V2 (that is, the volume fraction of the reinforcement phase in the TiB/Ti-6Al-4V composite is equal to 2%) as an example, the calculation of its equivalent stiffness matrix is briefly described. The equivalent material stiffness matrix of the RVE indicated in Equation (11) can be obtained through calculation based on the homogenization method mentioned previously. It is well known that the flexibility matrix for the orthogonal anisotropic material, ***S***, can be described by Equation (13) [33], which is inversely related to its stiffness matrix; that is, [***S***] = [***L***]^−^^1^. Therefore, by comparing Equations (12) and (13), the equivalent elastic parameters of the DRTMC can be acquired, as listed in Table 2.

(12) $$\left[L\right]=\left[\begin{array}{ccc} 1.301\times 10^{2} & 4.379\times 10^{1} & -7.539\times 10^{-3}\\ 4.379\times 10^{1} & 1.298\times 10^{2} & -7.131\times 10^{-3}\\ -7.539\times 10^{-3} & -7.131\times 10^{-3} & 4.307\times 10^{1} \end{array}\right]$$

(13) $$\left[S\right]=\left[\begin{array}{ccc} \frac{1}{E_{1}} & -\frac{\mu_{21}}{E_{2}} & 0\\ -\frac{\mu_{12}}{E_{2}} & \frac{1}{E_{2}} & 0\\ 0 & 0 & \frac{1}{G_{12}} \end{array}\right]$$

where *E_i_* is the elastic modulus, *μ_ij_* is the Poisson’s ratio, and *G_ij_* is the shear modulus.

Table 2 displays all of the calculated equivalent elastic parameters from V2, V3.5, and V5, together with the results from the literature [13]. It can be observed that the calculated *E*_1_ is quite close to *E*_2_ for each case, corresponding to different volume fractions. Therefore, the present composite can be regarded as isotropic material. Furthermore, this signifies that the generated RVE model may be reasonable, as it contains sufficient reinforcement phase material. Moreover, from Table 2, we can observe that, compared to the experimental results, the calculated equivalent elastic modulus for all cases, namely (*E*_1_ + *E*_2_)/2, exhibits a slight underestimate. However, the maximum error of these three cases is only 2.3%, which is acceptable in engineering applications. From these calculated equivalent elastic parameters, it can also be observed that the elastic module increases with the volume fraction of the reinforcement phase, but the Poisson’s ratio *μ*_12_ decreases. Therefore, all of the conclusions mentioned above fulfill our expectations. This implies that the developed modeling and analysis methods, as well as the generated RVE models, are reasonable and effective.

**Figure 5:**
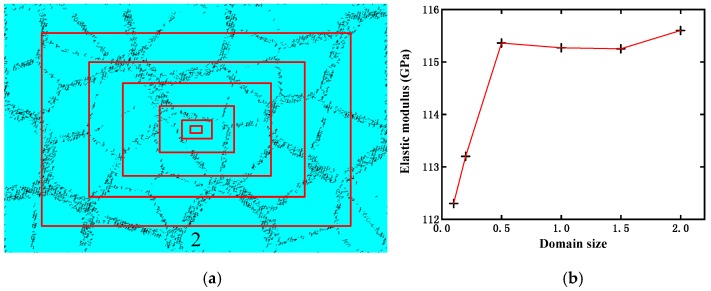
Size convergence study of RVE: (**a**) representative volume elements (RVEs) with different domain sizes; and (**b**) effective elastic modulus calculated from different RVE domain sizes.

Obviously, if the RVE is excessively small, it cannot represent the composite material behavior. However, an overly large RVE will lead to significant computational expenses and waste time. Although the generated RVE, including its size, appears reasonable according to the above discussion, its size convergence study was further conducted in our work. Firstly, a sufficiently large composite geometrical model was generated using our method, in which the same configuration and size of the microstructure as the actual material were considered. Thereafter, the domain size was changed to generate different sizes of RVE models, as illustrated in Figure 5a, for ultimately calculating their respective effective elastic modulus, as indicated in Figure 5b. In this case, the domain sizes 0.1, 0.2, 0.5, 1, 1.5, and 2 correspond to the actual sizes of the representative volume elements (RVEs) along the horizontal direction: 0.0639, 0.1278, 0.3194, 0.6388, 0.9581, and 1.2775 mm, respectively. In Figure 5b, it can be observed that the calculated effective elastic modulus tends to be stable when the domain size is larger than 0.5. Accordingly, in the following analyses, the RVE domain size was taken as 1.

## 5. Investigation on DRTMC Strengthening Mechanism

### 5.1. Influences from Simplification of Reinforcement Phase

**Figure 6:**
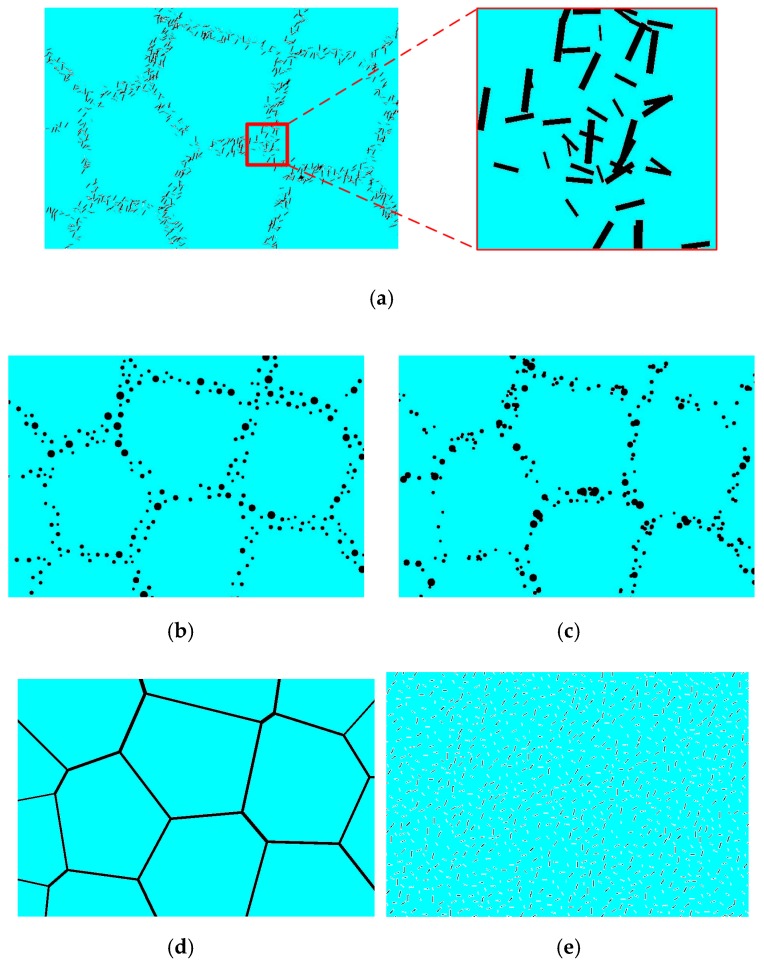
Five types of geometrical models used for comparative study: (**a**) Intersected rectangular particles; (**b**) separated circular particles; (**c**) intersected circular particles; (**d**) complete connectivity structure; and (**e**) uniformly distributed particles.

The reinforcement phase is an important component in the composite, which will significantly affect the overall composite mechanical properties. Because the configuration of the DRTMC reinforcement phase is extremely complex, it needs to be simplified for simulation. However, different simplifications will generally lead to varying results [34]. To ensure that the simulation results are reliable, five different types of simplified RVEs were generated and investigated. Figure 6 illustrates the geometrical models of the simplified RVEs with a particle content of 5%. In Figure 6a, the first model (Model I) is depicted, in which the reinforcement phase was approximated by using rectangular particles, and all of the particles can intersect with one another. In the second network-like model (Model II) illustrated in Figure 6b, the reinforcement phase was approximated by using different diameters of separated circular particles. This signifies that all of the particles do not overlap with one another. Figure 6c illustrates the third network-like model (Model III), in which the reinforcement phase was still approximated by using different diameters of the circular particles, but they can intersect with one another. Figure 6d describes the fourth network-like model (Model IV), in which the reinforcement phase was simplified as several different widths of straight lines, and these connect to one another completely, without any discontinuities. It should be noted that this model is an extreme case that can exhibit a superior load capacity. Figure 6e illustrates the uniformly distributed model (Model V), in which the reinforcement phase was approximated by using rectangular particles, and all of the particles can intersect with one another.

**Figure 7:**
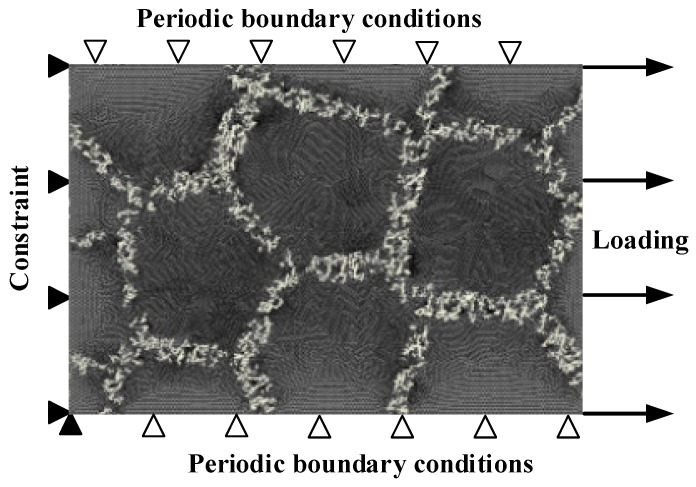
Finite element RVE model of DRTMC and its boundary conditions.

During the numerical simulations of the above five model types, the same settings as in the previous verification were considered, including the material constitutive relationship and its associated material parameters. A 2D RVE model of the DRTMC and its boundary conditions are illustrated in Figure 7. Following the simulations, both the instantaneous reaction forces and displacements for all nodes at the right boundary of RVE were extracted, as well as its geometrical information including the varying area and length. After homogenization treatment on all the nodes, both the instantaneous stresses and strains could be calculated and the true nonlinear stress–strain response curve could be obtained accordingly. The reasonableness of the 3D model simplified to a 2D model will be discussed further later.

**Figure 8:**
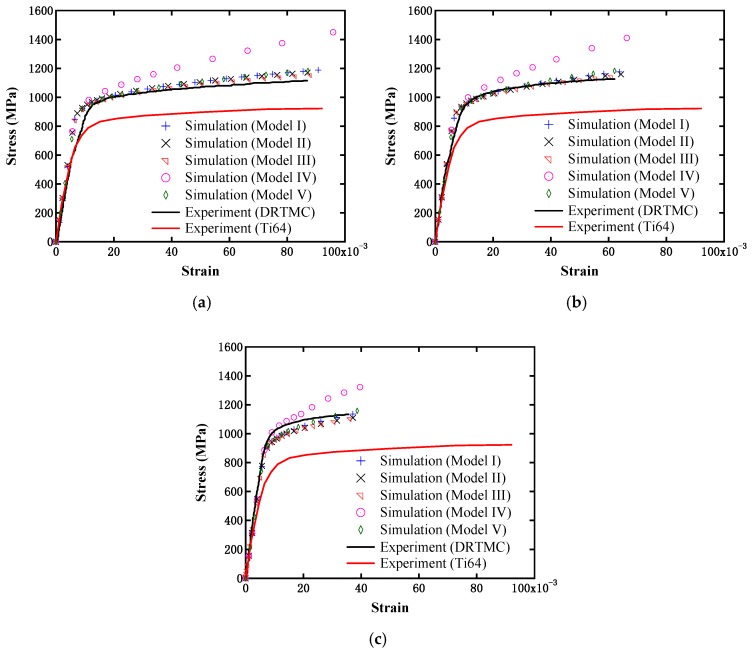
Comparison of stress–strain curves from simulations and experiments [13] with different particle contents: (**a**) 2%; (**b**) 3.5%; and (**c**) 5%.

In our work, the nonlinear true stress–strain response curves for V2, V3.5, and V5, as illustrated in Figure 8, were obtained for the comparative study. Different simplified shapes may exhibit different degrees of local stress concentration, but from a holistic perspective (owing to homogenization), it can be observed from this figure that all of the simulated curves, except for Model IV, were quite similar, and their amplitudes were also close to one another. Even in the extreme case, namely Model IV, its true stress–strain response curve at the initial yield stage was not substantially different from the other cases, and only its stress saturation segment exhibited a certain difference. As the reinforcement phase material is assumed to be linearly elastic, Model IV will result in higher stress, and the actual situation will be lower than the simulation result. Therefore, it can be inferred that simplification of the reinforcement phase has no significant effect on the overall material strengthening in the present DRTMC type. Thus, Model I is mainly discussed in the following investigation.

### 5.2. Load-Transferring Mechanism

As discussed previously, the literature studies demonstrate that the DRTMC strengthening mechanism is mainly attributed to two factors: the load-transferring mechanism between the reinforcement phase and matrix, and the strengthening caused by the metal matrix owing to grain refinement and the solid solution, among others. In our work, the load-transferring mechanism was first investigated through theoretical calculations.

**Figure 9:**
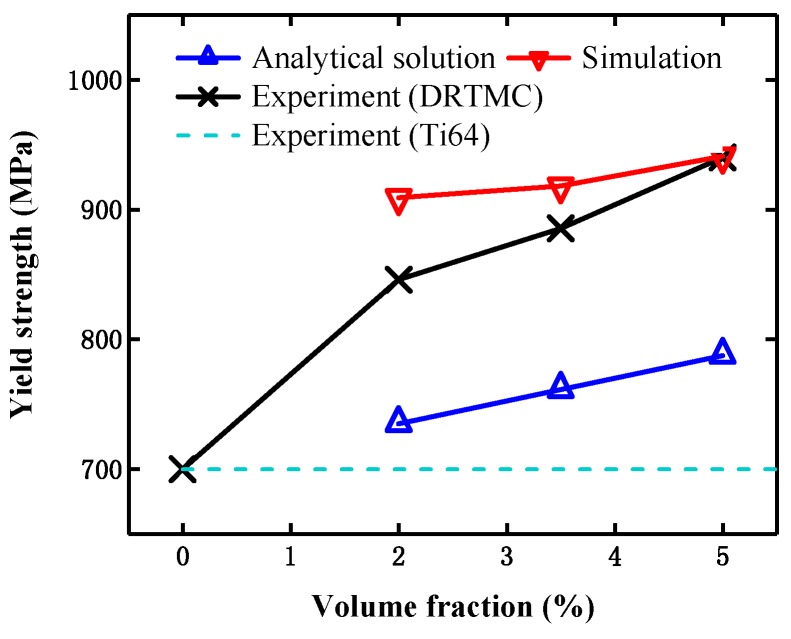
Comparison of effective yield strength obtained from simulation, analytical solution, and experiment [13].

In previous literature, the composite strength enhancement owing to the load-transferring mechanism was usually investigated through the generalized shear lag model, which was derived from the theory considering perfect bonding between the matrix and reinforcement phase, as follows [35]:

(14) $$\sigma_{cy}=\sigma_{my}[V_{e}\left(S+4\right)/4+V_{m}],$$

where *σ_cy_* is the composite yield strength, *σ_my_* is the matrix yield strength, *V_e_* is the content of the reinforcement phase, *S* is the aspect ratio of particles in the reinforcement phase, and *V_m_* is the content of the matrix. Actually, the present analytical formula itself does not consider the effect of the particle distribution forms in composites. Therefore, it was also applied to predict the yield strength of the composites with network-like microstructure, (e.g., in [36,37,38]). In our work, the aspect ratio of particles was taken as that provided in [13], namely *S* = 10, while the other parameters were taken as the previously mentioned values. In Figure 9, the effective yield strengths are compared, which were obtained from the analytical solution based on the load-transferring mechanism, the simulation results from Model I, and the experimental results from both the present composite material and its pure matrix phase (Ti64), respectively. As the present composite material had no obvious yielding process, the initial yield strength was defined as the stress value corresponding to 0.2% of the residual deformation. It can be observed from Figure 9, that the yield strengths obtained by the analytical solution were substantially smaller than the experimental values, but close to the yield strength of the matrix phase (see dashed line in Figure 9). It implies that the load-transferring mechanism has no obvious effect on the present composite material.

In general, compared to the uniformly distributed particle model (Model V), the network-like model (Model I) appears to possess a significantly stronger load-transferring capability. The reason is that the particles in the network-like model form a load-transferring channel much more easily, owing to nearer distances between particles and a higher probability of the particles merging. Therefore, in our work, a comparison between these two model types was further carried out to conduct an investigation. The simulation results are displayed in Figure 8. From this figure, it can be observed that the stress–strain curves exhibited little difference. Therefore, the strengthening owing to the load transferring between particles was also not obvious in the present composite. This may be a result of a lower volume fraction or distribution density of the reinforcement phase in the studied composite. Later, the influences of the volume fraction and distribution density of the reinforcement phases are discussed further.

### 5.3. Strengthening Mechanism of Matrix Material

In Figure 8, both the simulation results based on the adopted material constitutive relationship, and the experimental results [13] are presented. In the present material constitutive relationship, two hardening mechanisms (namely strain-gradient hardening and quench hardening) have been considered, which means that the strengthening mechanism of the matrix material could be embodied in the simulation. The effective yield strengths at three different particle contents of Model I were further extracted from their stress–strain curves in Figure 8, and are displayed in Figure 9. It can be observed from the above two figures that the simulation results were in good agreement with the experimental results, despite the effective yield strengths being slightly higher than the experimental values. Moreover, the present simulation results were substantially larger than those from the pure matrix material without considering the particle size effects. Therefore, it can be further verified that the DRTMC strengthening is mainly owing to the metal matrix strengthening.

### 5.4. Influences from Other Aspects

In addition to the above two mechanisms, our investigations also found that other factors, such as the distribution density and orientation of the reinforcement phase, will affect the DRTMC enhancement. In this paper, we explored such an influence from the above two factors in order to aid in further understanding the strengthening mechanism of the present composite material types. Furthermore, the reasonability of the 3D model simplified to a 2D model is discussed. As the enhancement phase is simplified to be linear and elastic, the calculated stress values are approximately larger than the real situation.

#### 5.4.1. Distribution Density of Reinforcement Phase

**Figure 10:**
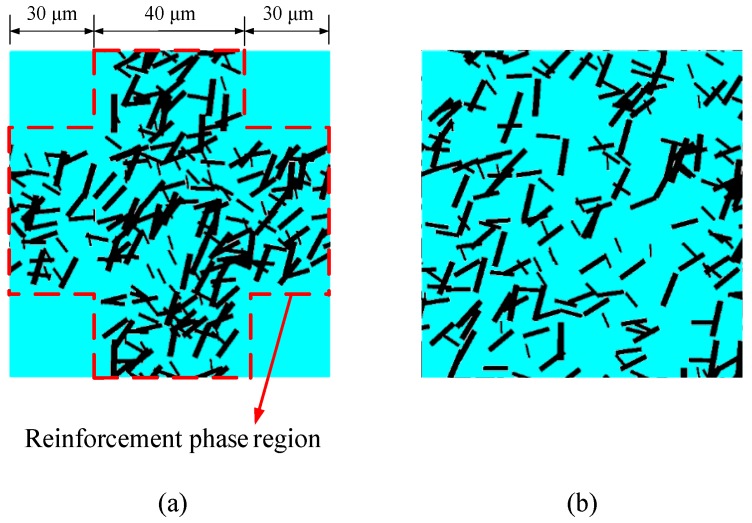
Small-scale RVE models with particles uniformly distributed in (**a**) cross-shaped region and (**b**) entire region.

Because the network-like RVE model illustrated in Figure 7 is too complex, it is not easy to reveal the differences in material properties caused by the reinforcement phase distribution. Hence, a smaller scale of RVE models, containing several reinforcement phases, was constructed to investigate the influence of the reinforcement phase distribution on material properties. In order to provide an improved explanation of the differences in the reinforcement phase distributions, and facilitate revealing the relevant mechanisms, we selected the following two RVE structures to conduct a comparative analysis. In the first structure (see Figure 10a), the representative microstructure in Figure 1, namely reinforcement particles distributed in a cross-shaped region, was modeled and studied. Finite element models with 25%, 38%, and 50% reinforcement phase contents were investigated, and five different random distributions for each were considered. In the second structure, the above three different contents of the reinforcement phases were uniformly dispersed throughout the matrix, while 15 sets of RVE models, considering random particle distributions, were constructed (see Figure 10b). The constitutive relations and boundary conditions of these two small-scale RVE models were consistent with those mentioned previously.

It is worth noting that there existed a significant difference in the density of the reinforcement phase, although the reinforcement phase contents for both models were maintained constant. For the network-like model illustrated in Figure 10a, the particle densities in the cross-shaped region were approximately 40%, 60%, and 80%, corresponding to the above three particle contents, respectively. However, for the uniformly distributed model illustrated in Figure 10b, the particle densities were equal to the respective particle contents; that is, 25%, 38%, and 50%.

**Figure 11:**
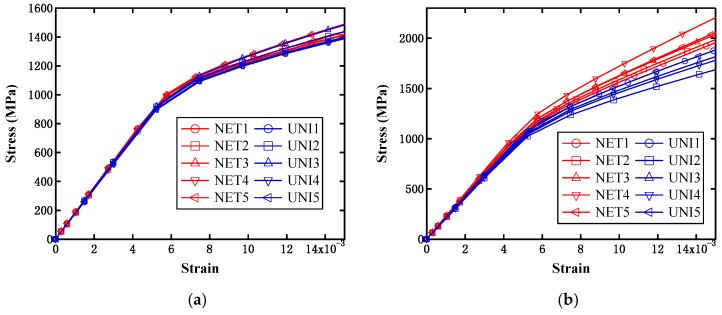

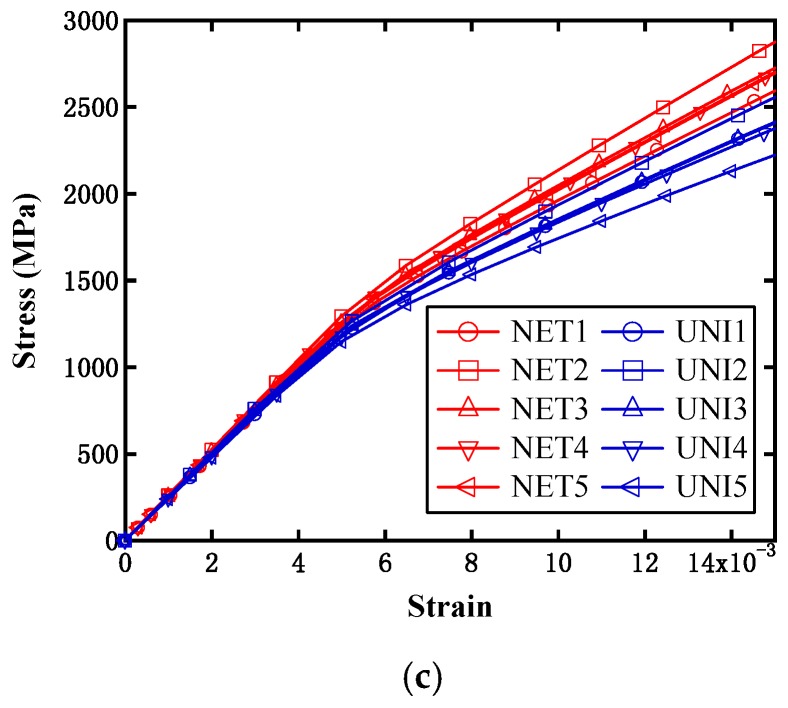
Simulated effective stress–strain curves from small-scale models with particle contents of (**a**) 25%; (**b**) 38%; and (**c**) 50%.

**Figure 12:**
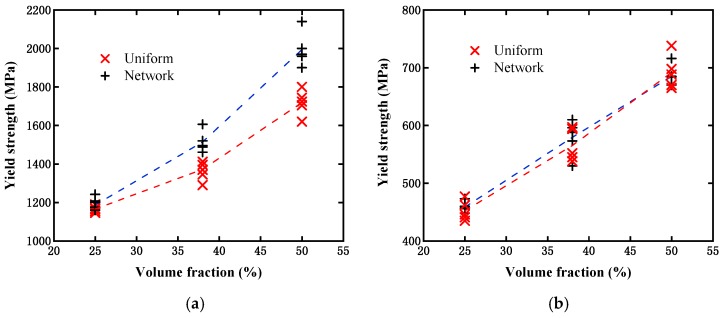
Comparison of initial yield strength evaluated from two models with different particle contents: (**a**) Under single axial tensile loading condition; and (**b**) under shear loading condition.

Figure 11 displays the simulated effective stress–strain curves from the above two models with different particle contents under the single axial tensile loading condition. NET1 to NET5 represent the simulation results from the five randomly distributed network-like models, while UNI1 to UNI5 denote the results from the uniformly distributed microstructures. It can be observed from the figure that the saturation stress increased with the particle contents, as expected, and all of the results were located within a narrow distribution range for both models, which signifies that such a random distribution of particles will not have a significant influence on the overall material performance. Moreover, it can be observed that the saturation stresses from both models were close to one another when the particle content was 25%. This implies that the effect on strength from the difference between the above two models can be neglected at a lower particle content. When the content was increased to 38%, the evaluated strength from the network-like model was obviously higher than that from the uniformly distributed model, and this trend became more obvious when the content reached 50%. It should be noted that in this case, all of the so-called initial yield strengths from the above curves were extracted in accordance with the rule of defining the initial yield strength for the case without containing an obvious yield process, and these are finally displayed in Figure 12a. This figure can aid us in more clearly observing such a difference between the two different models at varying particle contents. Moreover, we further investigated the difference between the above two different model types under the same shear loading conditions. From Figure 12b, it can also be observed that the initial yield strength increased with the particle content, but the difference between these two models could almost be ignored, which differs from the results based on the single axial tensile loading condition.

**Figure 13:**
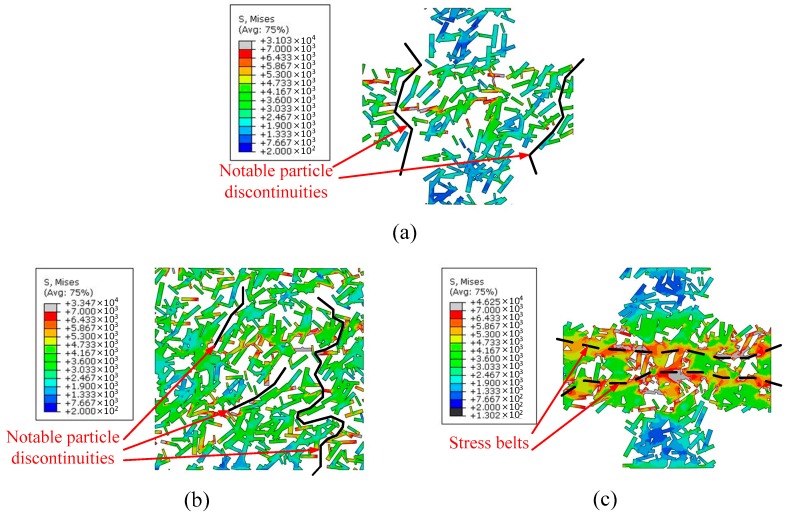
Reinforcement phase finite element Mises stress contours: (**a**) Network-like model with 25% particle content; (**b**) uniformly distributed model with 50% particle content; and (**c**) network-like model with 50% particle content.

As mentioned previously, it can be determined that the particle densities in the above models differed, although they had the same particle contents. Figure 13 illustrates the representative stress contours of the reinforcement phase in two models with two particle contents. Figure 13a,c display the network-like models with particle contents of 25% and 50%, respectively, while Figure 13b presents the uniformly distributed particle model with a particle content of 50%. Despite the differences between the models illustrated in Figure 13a,b, their particle densities were similar in the distribution region of the reinforcement phase. This signifies that these two models had a similar distribution form of the reinforcement phase. Accordingly, their average stress distribution ranges (see the change in color in the stress label) were close. Moreover, at this level of distribution density of the particles, one can easily observe an apparent feature, whereby several notable discontinuities (marked with black lines in Figure 13a,b occurred between particles although numerous particles connect to one another. This means that the load cannot be transferred by the stronger reinforcement phase at the locations with particle discontinuities. However, in the model presented in Figure 13c, two stress belts marked with black dashed lines can clearly be observed, in which the particles connect to one another without any discontinuities. Therefore, we know that the distribution density of the reinforcement phase is important for enhancing the composite strengthening. The higher particle distribution density signifies the increased probability of connections between particles, so that the load can easily be transferred from the particle channels, and the strength is therefore improved. Nevertheless, the particle content also has a certain influence on the material strengthening. It can be verified from Figure 12 that the initial yield strength in the uniform model with a particle content of 50% was larger than that in the network-like model with a particle content of 25%, although they had similar particle densities.

**Figure 14:**
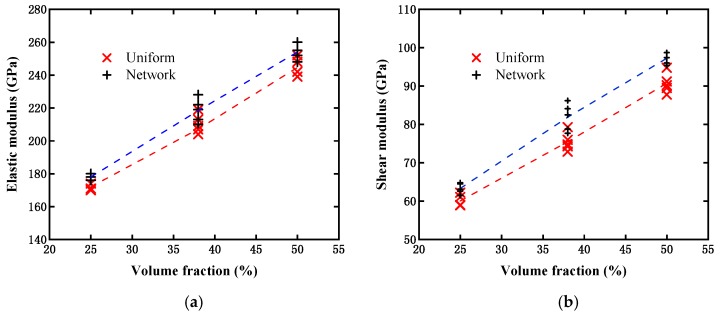
Comparison of modulus evaluated from two models with different particle contents: (**a**) Elastic modulus; and (**b**) shear modulus.

In our work, the effective elastic and shear moduli were also evaluated from the above two models with three different particle contents, and their results are illustrated in Figure 14. From the calculated data, it can be observed that the effective elastic and shear moduli for both models became larger as the particle content increased, which fulfills our expectation. However, the influences of the two different models on the elastic and shear moduli were slightly different from those of the yield strength. Both the effective elastic and shear moduli evaluated from the network-like model were slightly higher than those from the uniformly distributed particle model. For the single axial tensile loading case, the difference in yield strengths between the two models appeared larger than that of the elastic modulus. This can mainly be attributed to the elastic modulus of the reinforcement phase being not significantly larger than that of the matrix material in the present study, while the reinforcement phase in this study was presumed as elastic, in which state the reinforcement phase stress may be significantly higher than the yield stress of the matrix material. Therefore, the yield strength enhancement was more apparent than the elastic modulus in the present composite, as the distribution density of the reinforcement phase increased. For the shear loading case, the influence on the shear modulus was slightly larger than that on the yield strength. However, it still did not exhibit a significant difference from a statistical viewpoint.

#### 5.4.2. Distribution Orientation of Reinforcement Particles

**Figure 15:**
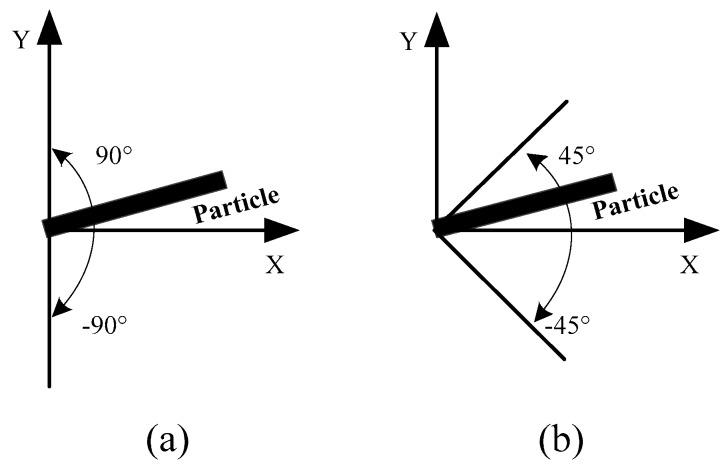
Schematic of distribution orientation of reinforced particle: (**a**) Randomly distributed at any orientation; and (**b**) randomly distributed at limited orientation.

As the distribution orientation of the reinforcement phase particles of the current composite material may have certain influence on the overall performance, the influence of the distribution orientation of the particles is discussed further here. In our work, the model with uniformly distributed rectangular particles was selected as the research object. The particle orientation was defined as the direction located with a larger size in the rectangular particle. The previous material properties were still adopted in these simulations. The loads in the horizontal (*x*-axis) and vertical (*y*-axis) directions were implemented to obtain the mechanical responses along these two directions. Figure 15 presents a schematic regarding two cases with different particle distributions in terms of their orientations. Case I consisted of particles uniformly distributed at any orientation, as illustrated in Figure 15a, while Case II consisted of particles uniformly distributed at a limited orientation (from −45° to 45° around the *x*-axis), as illustrated in Figure 15b.

**Figure 16:**
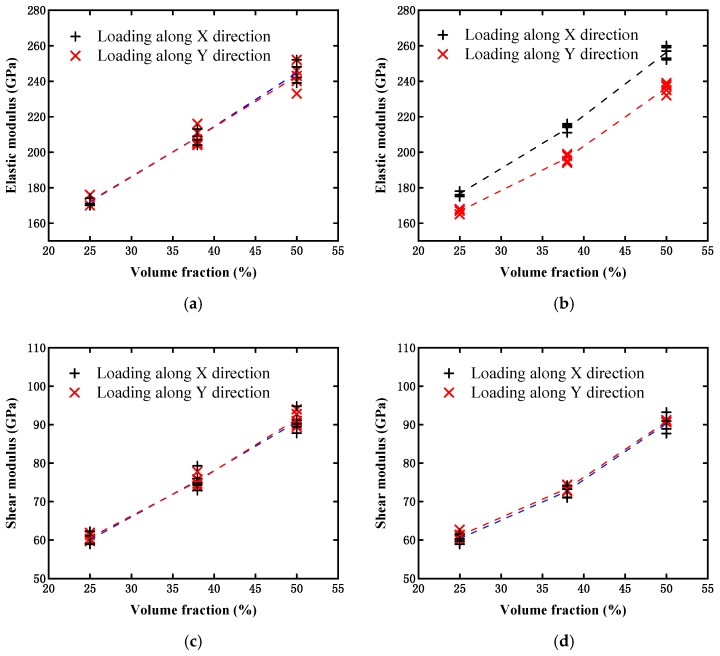
Comparison of moduli between non-limited (Case I) and limited (Case II) particle orientations: (**a**) Elastic modulus estimated from Case I under single axial loading; (**b**) elastic modulus estimated from Case II under single axial loading; (**c**) shear modulus estimated from Case I under pure shear loading; and (**d**) shear modulus estimated from Case II under pure shear loading.

Following the simulations, the evaluated effective elastic and shear moduli for the different distribution orientations of the particles are illustrated in Figure 16. Figure 16a,b present comparisons of the effective elastic modulus obtained from Case I and Case II, respectively. It can be observed that the elastic modulus in Case I increased with the particle content, but there was almost no difference in the results with the same particle content in the two loading cases. This indicates that the model without considering the limitation of the particle distribution orientation exhibited an overall isotropic feature of the material. However, for Case II, a prominent difference could be observed in the elastic modulus evaluated from the two-direction loadings, and *E_x_* was larger than *E_y_* when the particle orientation was limited within the range from −45° to 45° around the *x*-axis. The reason for this can be determined according to the following analysis. Along the *x*-axis direction, the RVE model in Case II had more reinforcement particles, owing to the limitation of the distribution orientation. The fact that more reinforcement particles existed in this direction signifies a higher probability of particles connecting to one another and forming load-transferring channels. Therefore, the composite could withstand a substantially larger load along this direction. In contrast, the material performance in the *y*-axis direction was weakened. Moreover, by comparing the magnitudes of the elastic modulus from Figure 16a,b, it could further be observed that the elastic modulus from Case I was located between *E_x_* and *E_y_* from Case II, for a model with a lower or higher particle content.

In addition to the single axial tension loading mentioned above, a comparison under the pure shear loading condition was also conducted between the non-limited and limited particle orientations. Figure 16c,d display the estimated shear modulus values from Case I and Case II, respectively. For each case, two shear loads, which were respectively applied at different surfaces along the x and y directions, were considered. It can be observed that all of the shear moduli increased with the particle content, and different loading forms would result in a negligible difference. However, for the medium content, the shear modulus values (both *G_x_* and *G_y_*) in Case II may be smaller than those in Case I. It can be understood that the limitation of the particle orientation signifies a lower intersection probability between particles. Therefore, for the medium particle contents, although from a statistical viewpoint slightly more particle materials were located along the *x*-axis than the *y*-axis, the probability of particles intersecting with one another in Case II may be lower than that in Case I, which would result in both shear moduli (*G_x_* and *G_y_*) in Case II being smaller than those in Case I. This implies that material strengthening owing to intersections between particles will be higher than that with slightly more reinforcement particles.

**Figure 17:**
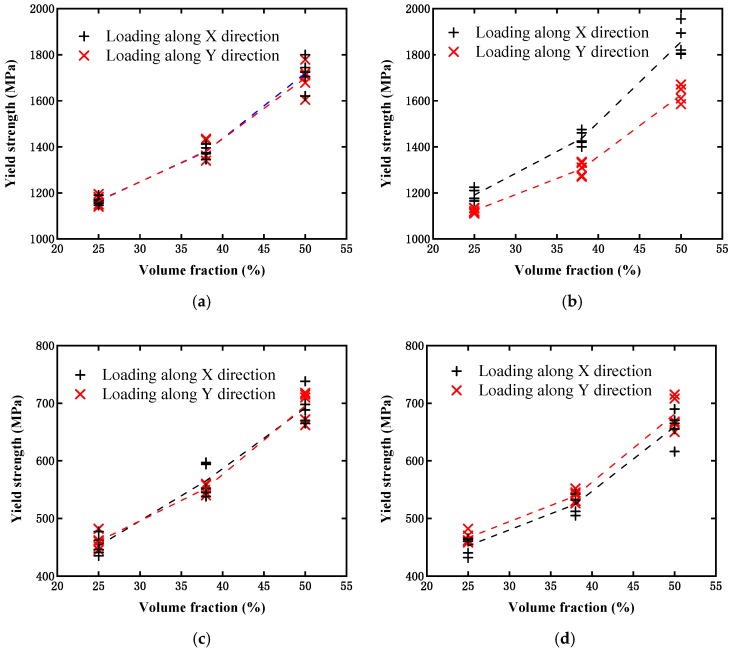
Comparison of effective yield stresses between non-limited (Case I) and limited (Case II) particle orientations: (**a**) Estimated from Case I under single axial loading; (**b**) estimated from Case II under single axial loading; (**c**) estimated from Case I under pure shear loading; and (**d**) estimated from Case II under pure shear loading.

Figure 17 illustrates the estimated effective yield stresses at both the non-limited and limited particle orientations, and both the single axial and pure shear load cases. All of the variation trends were quite close to those embodied in the elastic and shear modulus displayed in Figure 16, except for Figure 17d, which presents a comparison of the yield stresses between two different shear loads for the case with limited particle orientation. The loading along the vertical direction (*y*-axis) obviously resulted in a slightly higher effective yield stress than that in the horizontal direction (*x*-axis). This was caused by more particles intersecting along the horizontal direction. The presence of intersecting particles could hinder the possibility of matrix plasticity in the shear direction.

Therefore, a specific distribution orientation of particles will cause the composite to behave with the feature of anisotropy; that is, strengthening of mechanical performance at certain orientations and weakening at others.

#### 5.4.3. Influence of Simplification to 2D Model

The microstructure of the present composite is rather complicated, and it is nearly impossible to generate a 3D model perfectly mimicking the actual microstructure configuration. During the generation of the 3D finite element model, various issues will be encountered, such as failure to create an overlapping operation between different volumes, failure to generate the proper mesh owing to the existence of a small volume, and heavy computational costs. It is known that certain differences definitely exist between the 3D simulation and the 2D simulation simplified from the 3D model, particularly for the complicated composite material. Here, the differences between these two simulation types are further investigated. From the previous discussions, it can be concluded that the volume fraction of particles is the key influencing factor on the material properties of the present composite. Although the distribution form of the particle would also contribute a certain difference, it would not intrinsically change the mechanical behavior of the present composite. Therefore, the 3D modeling was simplified according to the following principles. Firstly, the particle volume fraction was maintained the same as the experimental data and that adopted in the 2D simulation. Secondly, relatively larger particles were considered in the 3D model. If the particle was excessively small, it would result in numerous modeling issues, which may cause abortion of the modeling.

**Figure 18:**
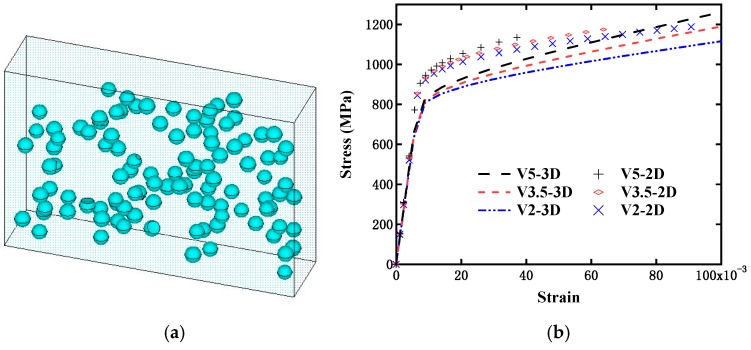
Influence of simplification to two-dimensional (2D) model: (**a**) Simplified three-dimensional (3D) RVE model of DRTMC; and (**b**) comparison of simulation results between 2D and 3D models with different particle volume fractions.

In our work, the previous three materials with different particle volume fractions were modeled, as illustrated in Figure 18a. Here, the same settings and conditions as in the previous simulations were adopted. The simulation results based on the 3D modeling, together with those from the 2D models, are displayed in Figure 18b. It can be observed that the overall trends varying with the volume fraction were similar for the 2D and 3D models, and their stress levels were also close, except for the initial yielding stresses being somewhat underestimated for the 3D cases. This may be caused by the simplification from the 3D model to the 2D model, or the simplified operations in the 3D modeling. However, such simplifications will not produce a significant influence on the characterization of the present composite material properties. Therefore, the previous discussions based on 2D simulations are reasonable and useful for aiding us in recognizing the strengthening mechanism of the present composite, and provide a valuable reference for the structural design of the present composite type.

## 6. Conclusions

In order to disclose the DRTMC strengthening mechanism, a novel modeling approach was developed, and implementation of the homogenization theory in ABAQUS was introduced. Ultimately, different microstructure models were generated, and the modified TNT model considering two strengthening mechanisms was used to investigate the influences of the load-transferring and strengthening mechanisms of the matrix material on the DRTMC mechanical response. The main conclusions can be drawn as follows.

(1) The present developed modeling method using the Thiessen polygon can realistically represent the network-like microstructure feature of the DRTMC. It can also easily be extended to describe other similar composite microstructures, and provides valuable insights into the optimization and design of this type of composite material.

(2) Based on the commercial finite element software ABAQUS and the developed user-defined subroutines, AMPBC can be conveniently applied in the RVE finite element model. With the aid of the homogenization method and the present implementation scheme in ABAQUS, the equivalent elastic parameters of the composites can be calculated effectively.

(3) The comparisons of simulation results from several simplified network-like distributed models indicates that the different simplifications of the reinforcement phase will not have any significant influences on the overall mechanical response of the studied DRTMC.

(4) The simulated results based on the present material constitutive relationship exhibit good agreement with the experimental results. By comparing the numerical predictions with the experimental data and analytical solution, it can be concluded that the strengthening of the matrix phase is the main cause of the overall strengthening of the present DRTMC material.

(5) By comparing the simulation results based on the models with different distribution forms of the reinforcement phase, including the distribution density and orientation, it is found that the specific distribution forms will also result in the strengthening effect, including producing the anisotropy feature, when the particle contents reach a certain threshold.

(6) For the studied composite with a lower volume fraction of reinforcement particles, the operation regarding simplification to the 2D model will not have a significant influence on the related factors.

## Figures and Tables

**Table 1:** Material properties of discontinuously-reinforced titanium matrix composite (DRTMC).

Materials	Elastic Modulus *E* (GPa)	Poisson’s Ratio *μ*	Initial Yield Stress *A* (MPa)	Strain Hardening Coefficient *K* (MPa)	Hardening Exponent *n*
Ti-6Al-4V	112.3	0.34	700	482.8	0.28
TiB	450	0.15	-	-	-

**Table 2:** Comparison of calculated equivalent elastic parameters of DRTMC.

Configuration	*E* (GPa)	*E*_1_ (GPa)	*E*_2_ (GPa)	*μ*_12_	$\frac{\left(E-\left(E_{1}+E_{2}\right)/2\right)}{E}$ (%)
V5-Experiment	122.5	-	-	-	2.3
V5-Simulation	-	119.7	119.6	0.332	
V3.5- Experiment	119.2	-	-	-	1.6
V3.5- Simulation	-	117.2	117.3	0.335	
V2- Experiment	116.0	-	-	-	0.9
V2- Simulation	-	115.0	115.0	0.337	

## References

[1] Gaisin R.A., Imayev V.M., Imayev R.M. (2017). Effect of hot forging on microstructure and mechanical properties of near α titanium alloy/TiB composites produced by casting. J. Alloys Compd..

[2] Lagos M.A., Agote I., Atxaga G., Adarraga O., Pambaguian L. (2016). Fabrication and characterisation of titanium matrix composites obtained using a combination of self propagating high temperature synthesis and spark plasma sintering. Mater. Sci. Eng. A.

[3] Li S.F., Kondoh K., Imai H., Chen B., Jia L., Umeda J. (2015). Microstructure and mechanical properties of P/M titanium matrix composites reinforced by in-situ synthesized TiC-TiB. Mater. Sci. Eng. A.

[4] Chandravanshi V.K., Sarkar R., Ghosal P., Kamat S.V., Nandy T.K. (2010). Effect of minor additions of boron on microstructure and mechanical properties of as-cast near α titanium alloy. Metall. Mater. Trans. A.

[5] Das M., Bhattacharya K., Dittrick S.A., Mandal C., Balla V.K., Kumar T.S.S., Bandyopadhyay A., Manna I. (2014). In situ synthesized TiB-TiN reinforced Ti6Al4V alloy composite coatings: Microstructure, tribological and in-vitro biocompatibility. J. Mech. Behav. Biomed. Mater..

[6] Wang J., Guo X., Qin J., Zhang D., Lu W. (2015). Microstructure and mechanical properties of investment casted titanium matrix composites with B4C additions. Mater. Sci. Eng. A.

[7] Zherebtsov S., Ozerov M., Stepanov N., Klimova M., Ivanisenko Y. (2017). Effect of high-pressure torsion on structure and microhardness of Ti/TiB metal-matrix composite. Metals.

[8] Guo X., Wang L., Wang M., Qin J., Zhang D., Lu W. (2012). Effects of degree of deformation on the microstructure, mechanical properties and texture of hybrid-reinforced titanium matrix composites. Acta Mater..

[9] Wang P., Nian G.D., Qu S.X., Shan Y., Hunag L., Peng H. (2017). Numerical study on mechanical properties of discontinuously reinforced titanium matrix composite with network reinforcement architecture. Int. J. Appl. Mech..

[10] Aradhya K.S.S., Doddamani M.R. (2015). Characterization of mechanical properties of SiC/Ti-6Al-4V metal matrix composite (MMC) using finite element method. Am. J. Mater. Sci..

[11] Giannopoulos G.I., Karagiannis D., Anifantis N.K. (2007). Micromechanical modeling of mechanical behavior of Ti–6Al–4V/TiB composites using FEM analysis. Comput. Mater. Sci..

[12] De Baere D., Bayat M., Mohanty S., Hattel J. (2018). Thermo-fluid-metallurgical modelling of the selective laser melting process chain. Procedia CIRP.

[13] Huang L.J., Geng L., Peng H.X., Zhang J. (2011). Room temperature tensile fracture characteristics of in situ TiB/Ti6Al4V composites with a quasi-continuous network architecture. Scr. Mater..

[14] Christofferson R., Nilsson O. (1989). Placentation in the rat: A sem study of microvascular casts. Prog. Clin. Biol. Res..

[15] Fritzen F., Thomas B. (2011). Nonlinear homogenization using the nonuniform transformation field analysis. PAMM.

[16] Wimmer J., Schnepp J., Reese S. (2015). Modelling and Simulation of Asphalt. PAMM.

[17] Voronoi G. (1908). Nouvelles applications des paramètres continus à la théorie des formes quadratiques. Deuxième mémoire. Recherches sur les parallélloèdres primitifs. J. Reine Angew. Math..

[18] Qu S., Siegmund T., Huang Y., Wu P.D., Zhang F., Hwang K.C. (2005). A study of particle size effect and interface fracture in aluminum alloy composite via an extended conventional theory of mechanism-based strain-gradient plasticity. Compos. Sci. Technol..

[19] Su Y., Ouyang Q., Zhang W., Li Z., Guo Q., Fan G., Zhang D. (2014). Composite structure modeling and mechanical behavior of particle reinforced metal matrix composites. Mater. Sci. Eng. A.

[20] Cao G., Geng L., Naka M. (2006). Elastic properties of titanium monoboride measured by nanoindentation. J. Am. Ceram. Soc..

[21] Panda K.B., Chandran K.S.R. (2006). First principles determination of elastic constants and chemical bonding of titanium boride (TiB) on the basis of density functional theory. Acta Mater..

[22] Madtha S., Lee C., Ravi Chandran K.S. (2008). Physical and mechanical properties of nanostructured titanium boride (TiB) ceramic. J. Am. Ceram. Soc..

[23] Arsenault R.J., Wang L., Feng C.R. (1991). Strengthening of composites due to microstructural changes in the matrix. Acta Metall. Mater..

[24] Arsenault R.J., Shi N. (1986). Dislocation generation due to differences between the coefficients of thermal expansion. Mater. Sci. Eng..

[25] Nan C.W., Clarke D.R. (1996). The influence of particle size and particle fracture on the elastic/plastic deformation of metal matrix composites. Acta Mater..

[26] Shen X.J., Zhang C., Yang Y.G., Liu L. (2019). On the microstructure, mechanical properties and wear resistance of an additively manufactured Ti64/metallic glass composite. Addit. Manuf..

[27] Fukuhara M., Sanpei A. (1993). Elastic moduli and internal frictions of Inconel 718 and Ti-6Al-4V as a function of temperature. J. Mater. Sci. Lett..

[28] Gao H., Huang Y. (2001). Taylor-based nonlocal theory of plasticity. Int. J. Solids Struct..

[29] Gao X., Zhang X., Geng L. (2019). Strengthening and fracture behaviors in SiCp/Al composites with network particle distribution architecture. Mater. Sci. Eng. A.

[30] Xia Z.H., Zhou C.W., Yong Q.L., Wang X. (2006). On selection of repeated unit cell model and application of unified periodic boundary conditions in micro-mechanical analysis of composites. Int. J. Solids Struct..

[31] Yuan Z., Fish J. (2008). Toward realization of computational homogenization in practice. Int. J. Numer. Methods Eng..

[32] Xu Y.J., Wu P.W., Zhao S., Wang X., Liang L. (2017). Implementation of elastic-plastic multi-scale analysis and application in particle reinforced composites. Acta Mater. Compos. Sin..

[33] Oliveira J.A., Pinho-da-Cruz J., Teixeira-Dias F. (2009). Asymptotic homogenisation in linear elasticity. Part II: Finite element procedures and multiscale applications. Comput. Mater. Sci..

[34] Williams J.J., Segurado J., LLorca J., Chawla N. (2012). Three dimensional (3D) microstructure-based modeling of interfacial decohesion in particle reinforced metal matrix composites. Mater. Sci. Eng. A.

[35] Nardone V.C. (1987). Assessment of models used to predict the strength of discontinous silicon carbide reinforced aluminum alloys. Scr. Metall..

[36] Koo M.Y., Park J.S., Park M.K., Kim K.T., Hong S.H. (2012). Effect of aspect ratios of in situ formed TiB whiskers on the mechanical properties of TiBw/Ti–6Al–4V composites. Scr. Mater..

[37] Hu Z.Y., Cheng X.W., Li S.L., Zhang H.M., Wang H., Zhang Z.H., Wang F.C. (2017). Investigation on the microstructure, room and high temperature mechanical behaviors and strengthening mechanisms of the (TiB+TiC)/TC4 composites. J. Alloys Compd..

[38] Liu B.X., Huang L.J., Geng L., Wang B., Cui X.P. (2014). Effects of reinforcement volume fraction on tensile behaviors of laminated Ti–TiBw/Ti composites. Mater. Sci. Eng. A.

